# Population Data Centre Profiles: Centre for Data Linkage

**DOI:** 10.23889/ijpds.v4i2.1139

**Published:** 2020-03-11

**Authors:** JH Boyd, SM Randall, AP Brown, M Maller, D Botes, M Gillies, A Ferrante

**Affiliations:** 1 Centre for Data Linkage, School of Public Health, Curtin University; 2 Department of Public Health, School of Psychology and Public Health, College of Science, Health & Engineering, La Trobe University

## Abstract

The Centre for Data Linkage (CDL) was established at Curtin University, Western Australia, to develop infrastructure to enable cross-jurisdictional record linkage in Australia. The CDL’s operating model makes use of the ‘separation principle’, with content data typically provided to researchers directly by the data custodian; jurisdictional linkage where available are used within the linkage process. Along with conducting record linkage, the team has also invested in establishing a research programme in record linkage methodology and in developing modern record linkage software which can handle the size and complexity of today’s workloads. The Centre has been instrumental in the development of practical methods for privacy-preserving record linkage, with this methodology now regularly used for real-world linkages. While the promise of a nation-wide linkage system in Australia has yet to be met, distributed models provide a potential solution.

## Background

The Centre for Data Linkage (CDL) is a component of the national Population Health Research Network (PHRN) and was established in 2009 within Curtin University as part of the National Collaborative Research Infrastructure Strategy funded by the Australian Government. The focus was to develop and implement secure, state-of-the-art infrastructure to enable large scale and cross-sectoral data linkage for research in Australia (1). The Centre has continued to receive funding via the PHRN, supplemented with competitive research and consultancy funding.

Australia’s federated government system means that various datasets are gathered at different tiers of administration that operate independently from one another. Linkage units exist in many states across Australia, primarily linking local, state-based health datasets (secondary and tertiary care). The PHRN initiative aims to provide cross-jurisdictional linkage, i.e. linkage of data across nine different legal jurisdictions (six States, two Territories and the Commonwealth Government of Australia). The full potential of these data resources can only be realised by linking data between these jurisdictions to ensure complete population coverage.

## Approach

A federated government coupled with a complex authorizing landscape has made a move to an enduring national linkage map difficult to achieve in Australia in the first instance. The platform established by the CDL has been used for a number of large research projects requiring linkage of different state and national datasets, including those requiring the establishment of a long-term linkage map. It is anticipated that this project-based approach will evolve and mature into an ongoing enduring national linkage model.

From the outset, the CDL focused on three key areas: i) carrying out record linkage for clients, ii) conducting research into record linkage methods, and iii) developing software and tools necessary for high quality record linkage. Each area is directly informed by and related to the others. Research questions are borne out of issues encountered during routine record linkage. Newly developed methodology published by the team is then implemented into the linkage software. The linkage software is then used operationally for routine linkage for clients. 

## Operating Model

The CDL is very aware of the sensitivities associated with maintaining databases containing identifiable information, utilising the ‘separation principle’ to link data from multiple jurisdictions [1]. This approach distinguishes between the data items used to link records (demographic data) and those items used by researchers to answer their specific questions (content data) [2].

Under the model adopted by the CDL, the linkage team are supplied with demographic data from participating datasets. This information is used to generate a Linkage Map. The Linkage Map is central to the CDL linkage model and consists of 'pointers' to records in the contributing data collections; the Map identifies the same person within and between datasets. Although the creation of the Map requires access to individually identifiable demographic information, these data items are stored separately from the Linkage Map. For privacy reasons, the linkers do not have access to the content data - this remains under the full control of the relevant dataset's custodian. Content data is released by the original data custodian (not the CDL) to the researcher for approved research projects, either directly to the researcher or to a secure analytical research environment (i.e. a safe-haven).

### Designing Secure Linkage Infrastructure

The development of a secure linkage infrastructure requires both secure linkage software to carry out the necessary functions, and a secure environment (including appropriate information governance) to host this software. In terms of an operating environment, the objectives of the CDL infrastructure are:

1. To provide secure linkage systems and services to internal and external stakeholders, with adequate levels of availability;
2. To provide an environment that is auditable and certifiable against the PHRN Information Governance Framework, and other industry standards;
3. To provide a cost effective, low maintenance environment that can draw on shared services within a provider’s managed environment (e.g. software updates, licencing, networks, directories, security technologies).

The infrastructure is designed to provide a platform for undertaking large linkage projects while meeting the requirement to provide a secure and controlled environment for working with sensitive data [3, 4].

The infrastructure supports the following core functions:

- Provision of demographic information from data custodians to the CDL;
- Linkage of this data to create project specific linkage keys;
- Supply of keys back to the various data custodians in each jurisdiction;

To future proof the secure data linkage facility, it was important to create scalable infrastructure that could accommodate both the increasing demand for linked data and the increasing volume and complexity of datasets to be linked.

### Information Governance

A challenge for the CDL was to translate information governance frameworks and standards into a set of rules, concepts and designs that could be implemented as a cost-effective technical solution [5]. The model development involved working with IT departments (or outsourced providers) whose priority and expertise is in supporting corporate systems (e.g. finance and HR), not necessarily dealing with the specialised needs of linkage researchers and analysts [6].

The design process involved developing a set of guidance infrastructure architectures or ‘Design and Implementation Guidelines’ for a secure research computing environment to host the CDL infrastructure [7, 8]. The design allows the CDL to store and use the data provided for each linkage project in a highly secure environment. This includes physical security features (such as key card access to the CDL office, additional card access for entry into secure computer rooms and a safe to store protected information in physical form e.g. DVD) as well as technical security measures (such as computers requiring password login, automatic screen locking and monitoring of login attempts) and data security (e.g. the use of encryption to store information). This environment was later assessed through an independent external security audit, which found no major deficiencies. More recently, the CDL has attained ISO 27001:2013 certification, a globally recognised information security management standard, making it one of the only parts of Curtin University and one of the few research groups in Australia to achieve this standard.

The CDL also obtained human research ethics committee (HREC) approval to establish the core operations of the linkage system, that is, the capacity to receive demographic data to generate a Linkage Map and to release linkage keys to providers. In addition to this approval, other state-based HREC approvals were obtained to allow construction of a Linkage Map for specific projects so that state and territory data providers could release their demographic data to the CDL for linkage. Researchers wishing to use the linked data created through this process required further, additional ethical approvals.

There are limited legislative barriers to record linkage in Australia. The overarching federal privacy legislation provides a mechanism for the release of data for health research with and without individual consent. Similar laws, with similar exemptions for research under condition, exist at the state level. However, there is also no obligation on data custodians to provide their data for linkage. A culture of risk aversion in government, particularly at the Commonwealth level, has existed in the past which has reduced access to government datasets for research [9]. The provision of data to a trusted third party to enable record linkage offers some benefit to data custodians, however this approach also increases the risk of data disclosure. Recent pushes from the top levels of government for enabling the sharing of data suggests that access to data for research may improve in the future [9].

### LinXmart: Development of secure, scalable linkage software

An early environmental scan and software evaluation by the CDL suggested few if any available linkage systems could provide a robust enterprise-grade platform which could easily scale to the data sizes anticipated for linkage across Australia, and which could manage the complexity of ongoing enduring linkage for multiple research projects [10]. As such, a decision was made to develop software to meet these needs. A linkage system was developed over a series of construction and release iterations, and user acceptance testing phases. As the software developed, the priorities of selected features were reviewed and re-assessed to ensure that software aligned to the business priorities of the CDL. New features and functions were regularly added to the software as the requirements of the CDL changed over time. The software is now used for all record linkage projects conducted by the CDL. More recently, the software has been utilised by a number of outside organisations for their linkage work.

### System requirements/features

At a basic level, the linkage software has been designed to undertake linkage across event-level datasets, based on fully configurable matching of demographic information using the standard Fellegi-Sunter probabilistic linkage approach. The software also performs data extraction to satisfy requests from jurisdictional data providers to supply encrypted project-specific identifiers for release to researchers [11].

To ensure that the linkage system was ‘fit for purpose’, the software was developed to include linkage and management capabilities. The system was designed using a component approach which focused on system integration, interoperability and expansion capabilities to ensure future flexibility. The ‘baseline’ development criteria included the following requirements:

#### Secure and auditable

Security was implemented in a role-based access control model. This method regulates access to the system based on the role of individual users. The system roles and their implementation were defined as part of the system architecture and are managed through standard operating procedures. User roles can be created, changed or withdrawn as the needs of the service change, without individually updating the privileges for every user. Administrative and monitoring functionality allows operators to manage linkage projects and data from different data providers. Linkage, quality assurance and data extraction processes are monitored through the user interface, and built-in audit trails track operations on all processes, data (records or transactions) and data custodian details.

#### Enduring and project linkage

The system manages a range of projects from a simple ‘one-off’ project with a short life span through to enduring longitudinal datasets that are constructed and updated through the ongoing linkage of records over time. The ability to manage both types of projects ensures flexibility and versatility in linkage operations.

#### Data volume

All system components (load, linkage, data management and output) have the capacity to handle large data volumes. The linkage projects carried out by the CDL regularly involve tens of millions of records and billions of matching transactions, and ensuring the software can manage these sizes is crucial.

#### Project management

The system is designed to manage multiple projects without performance overheads. The user interface provides operators with the ability to create and manage projects (linkage and extraction), custodians and data. The system can manage multiple large projects without substantial or complex operator involvement. The system is also designed to manage the process of data extraction for specific research projects. Project extractions are controlled by the system and produce a project-specific linkage map for the researcher. The project keys generated in the Map are only relevant to an individual linkage project (even if the dataset appears in another linkage). This ensures no cross-over between projects or project teams.

#### Link management

Unlike most linkage systems, the software has been designed to manage changes in data and links over time. The software can automatically process amended and deleted records as well as deal with new records. Unlike other designs, the system stores and processes all linkage transactions at the matching pair level. This allows the system to automatically detect and manage change to the linkage map as data is added (including new records and amendments to records). It also supports ‘any point in time’ referencing at the group or map level allowing operators to recreate the linkage structure for any records at any (previous) point in time.

The linkage software uses a layered architecture built on the .NET framework and uses Microsoft SQL Server for data storage. The system is managed through a web interface which allows role-based access to linkage functions and features. Linkage tasks are run on a single processing node that allows compute resources to be vertically scaled as required. The linkage tools and services are installed within a specifically designed secure environment (ISO27001 certified) hosted at Curtin University.

### From research to practice: privacy-preserving record linkage

The development of software also provided synergies with one of the other core functions of the CDL; to conduct research into record linkage methods. The development of our own software has enabled improvements in record linkage methods to be directly translated into software and then used in a real-world production environment. A key example of this is the development and use of techniques for privacy-preserving record linkage.

Privacy-preserving record linkage (PPRL) has been a major development in the record linkage arena internationally [12]. These techniques allow linkage to occur with only encoded personal identifiers available. Under optimal conditions, these methods can achieve the same quality as un-encoded linkage [13]. PPRL techniques may be particularly useful given the reticence of many custodians to provide person identifiers for linkage given privacy concerns. The CDL, along with a number of other groups, have played a key role in developing, implementing and popularising techniques allowing record linkage to occur on encoded personal identifiers [13-16].

Techniques for privacy-preserving linkage have been incorporated into CDL’s linkage software and a number of pilot and/or proof of concept projects have tested and evaluated the capabilities of privacy-preserving linkage in a real-world context [13, 17].

For these projects, the CDL has used the Bloom filter approach for privacy preserving record linkage. The process starts by encoding personally identifying information into Bloom filters (binary vectors). The method provides strong protection as the encoding process is irreversible and the encoded output is distorted to the extent that accidental recognition of an individual is impossible. The Bloom Filter encoding means that the matching can be carried out within the context of a traditional Fellegi-Sunter probabilistic linkage [13-16]. These techniques have now been deployed for project-based linkage, with the CDL using privacy-preserving record linkage methods for a number of real-world projects, including an NHMRC-funded project investigating the continuity of care provided by primary and secondary health services and a more recent project linking health and non-health records to create a Social Investment Data Resource (described below). Typically, these are linkages which otherwise would not have occurred without the availability of this technology.

The development of probabilistic linkage techniques that do not require the release of personal information but protect privacy through data encoding represents a significant breakthrough in data linkage methods. The application of these methods within operational linkage environments not only strengthens security but increases linked research opportunities as previously inaccessible datasets now become available for research. 

### Extending linkage beyond the boundaries of health: Social Investment Data Resource

A recent key project for CDL has been the development of the Social Investment Data Resource (SIDR). The concept of the SIDR is to create an integrated and accessible resource containing de-identified, unit-level data about a cohort of young people who come into contact with the Western Australian criminal justice system. The West Australian (WA) criminal justice sector (which includes the police, courts, corrections and juvenile justice) has no routine way of linking up core datasets with and across organisations. Therefore, it is not possible to investigate the impact of any intervention program on any targeted group.

Using the existing CDL architecture, the SIDR has been designed to be securely and strictly managed, providing secure access to authorised users through several mechanisms, including direct access and statistical analysis tools.

The SIDR contains population-level data sourced from WA birth registrations, school enrolment information and other datasets from police, justice, child protection, housing and education. The linkage model requires the release of relevant name-identified data from key agencies (e.g. Registrar of Births, Deaths and Marriages) to CDL. The CDL matches these records, to determine the same individual across multiple datasets.

To address legislative and privacy concerns, the SIDR linkage model has been designed to operate using a combination of clear text linkage and encoded data for privacy-preserving record linkage i.e. some of these linkages are conducted using personal identifiers, while privacy-preserving record linkage techniques are used in other circumstances.

Significantly, this project also incorporates a bifurcated data linkage model, where data linkage responsibilities are shared between the CDL and the WA Department of Health Data Linkage Branch (DLB). The CDL has primary responsibility for linkage of non-health datasets and for enabling the build of the SIDR, while the DLB continues to undertake the linkage of all health-related data and to service research through the provision of value-added services (like family connections) [18, 19].

## Discussion

The CDL has played a key role in Australian linkage infrastructure, and its linkage expertise is recognised nationally and internationally. Research into record linkage methods has resulted in over twenty peer-reviewed publications. The CDL has established a secure and efficient data linkage environment and developed innovative linkage software, utilized by CDL and a number of other organisations. The CDL initially focussed on large cross-jurisdictional and cross-sectoral linkage projects, which often involved tens of millions of records for a range of clients and research projects. More recently, the CDL has provided linkage services for health (e.g. registries and clinical systems) and non-health (e.g. education and criminal justice) projects. The CDL team is relatively small with 8-10 people included in the various linkage activities.

**Figure 1: An example of a distributed model. Each linkage unit receives the same spine file; they each link their own jurisdictions datasets to this spine, creating their own ‘master linkage key (MLK) file’.**
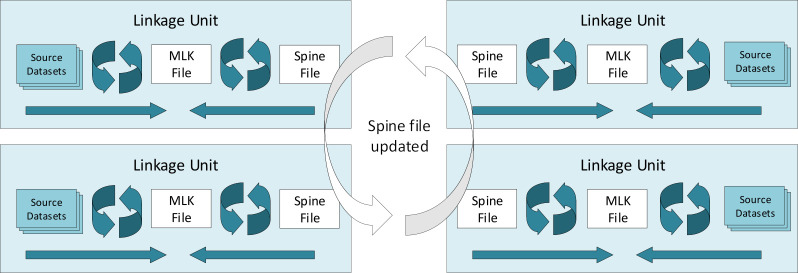


The CDL is also currently pioneering a distributed data linkage model with the DLB. Given the fragmented nature of health care service delivery in Australia, the impact of health funding, health service planning and health outcomes can only be achieved through efficient linkage/integration infrastructure. Experiences from other countries demonstrate the need to harness and harmonise the power and experience of linkage services and systems to improve the efficiency and quality within overall data linkage infrastructure. Given the numerous stakeholders and linkage services operating in Australia, the most promising path may not be to develop a single national linkage map managed by one organisation but to develop distributed models which utilize all available resources to achieve the same end goal.

A challenge in Australia is to realise the potential of the infrastructure currently available across government and university sectors through compatible, sustainable and effective models which can maximise the capacity across all these systems. The methods and techniques around data linkage in Australia are well established, and new developments (exploiting advances in technology) have the potential to improve access, timeliness and efficiency.

Challenges to realising the potential of data linkage in Australia remain. Increasing demand for data linkage services has put significant pressure on infrastructure to deliver in a timely fashion. The linkage community needs to focus on mechanisms which will ensure the timely delivery of data, particularly as the number, size and complexity of linkage research projects increase. Continuous improvement will also be essential. The infrastructure must continue to identify and implement new technologies to improve the efficiency of data linkage in Australia. Potential exists to improve the workflow and data flow between custodians, linkage units and researchers. Australia needs to ensure that data linkage infrastructure and technologies are interoperable and responsive to environmental changes around legislation, information technology, security and privacy.

### Distributed linkage

Although there has been considerable work to address the technical and methodological challenges associated with large-scale data linkage, the establishment of an enduring national linkage map has remained elusive. With linkage facilities in place across Australia, there are opportunities to leverage expertise and best practices. Development of interoperable systems would increase capacity and speed of data linkage processes. Partnerships between data linkage activities can drive innovation and efficiency within linkage systems.

One solution may be through a distributed data linkage model which utilises existing data linkage services available through multiple linkage units to facilitate the access and linkage of multiple datasets. A distributed linkage approach would enable participating linkage units to continue maintaining their existing master linkage key systems, as well as operating in an integrated way to facilitate enduring linkages. It would utilise existing skills and experience, standards, governance arrangements and infrastructure in participating linkage nodes. The model would both standardise existing linkage approaches and provide a scalable platform to capture capability across multiple linkage nodes.

A basic approach would involve the sharing across all linkage units of a common ‘spine’ file; a dataset or amalgamation of datasets which contains most of the population. Each linkage unit could then link their jurisdictions datasets to this spine independently. This would effectively result in a national linkage map, albeit one not held by any single party (see Figure 1). The model would allow linkage units to continue maintaining their linkage map as well as operate in an integrated way to facilitate enduring linkages across multiple linkage domains. Importantly, this distributed model would utilise the trust relationships developed between local linkage units and local data custodians, a key asset. Ultimately, it would provide timely integration of a greater range and diversity of linked datasets.

## Conclusion

The ultimate aim of any organisation dedicated to record linkage must be to improve access to and quality of linked data. Carrying out this mission will provide the community with a greater number of research outputs with greater confidence in their validity. The CDL has addressed this challenge in a number of ways. It has worked to carry out cross-jurisdictional and cross-sectoral linkage projects that would not have been possible otherwise. It has developed a research program dedicated to investigating best practice methods, and developing new methodologies for record linkage, with a strong focus on enhancing linkage quality and enabling data access. It has developed linkage software which meets the challenges of modern linkage units, which has been used not just to improve CDL’s processes, but by outside linkage organisations as well.

The research enabled by record linkage continues to improve lives through changes in health and social policy and clinical practice [20]. The investments made by the CDL will only further this. 

## Ethics statement

No ethical approval was sought for this publication.
